# Herpes Simplex Virus Encephalitis: Atypical Presentation as a Right Middle Cerebral Artery Stroke

**DOI:** 10.7759/cureus.2067

**Published:** 2018-01-15

**Authors:** Maria Shoaib, Jacqueline J Kraus, Muhammad T Khan

**Affiliations:** 1 Department of Medicine, Dow Medical College Pakistan; 2 Attending Physician, Charleston Area Medical Center / West Virginia University

**Keywords:** atypical presentation, encephalitis, herpes simplex virus, right middle cerebral artery, stroke

## Abstract

Herpes simplex virus encephalitis (HSVE) is a medical emergency associated with high mortality and morbidity. Definitive diagnosis is established by history, clinical examination, neuroimaging studies, supportive electroencephalogram (EEG) findings, and cerebrospinal fluid (CSF) analysis.

We report a case of HSVE presenting as a stroke mimic in a 76-year-old female with a history of atrial fibrillation on warfarin. She was admitted to our medical intensive care unit with intermittent fever, lethargy, and new onset left-sided hemiparesis. A computed tomography (CT) of the head showed a right middle cerebral artery (MCA) acute ischemic stroke with midline shift and a dense right MCA sign. Brain magnetic resonance imaging (MRI) showed evidence of acute stroke with consideration of herpes encephalitis. CSF analysis was positive for herpes simplex virus (HSV) type one. She recovered with high-dose intravenous acyclovir therapy.

Our patient was a diagnostic dilemma, initially being diagnosed with an acute ischemic stroke and yet found to have HSVE, which mimicked an acute ischemic stroke. Delay in treatment may result in devastating clinical outcomes that may include severe cognitive, focal neurological deficits, persistent seizures, and even death. This case highlights the importance of a multidisciplinary approach and the need for increased awareness of an atypical presentation of HSVE among emergency physicians, neurologist, intensivists, and radiologists.

## Introduction

Herpes simplex viral encephalitis (HSVE) is a neurological emergency with high morbidity and mortality. It is most commonly caused by herpes simplex virus type one (90%), followed by herpes simplex virus type two (10%). The presentation is usually nonspecific and may include acute febrile illness with headache, progressive altered mental status, hemiparesis, and seizures [1].

The virus shows neurotropism with a predilection for medial temporal and inferior frontal lobes. It induces latent or persistent infections via sensory neural pathways. The primary infection involves the mucocutaneous surfaces, which serve as the portal of entry of the viral particles into the nervous system within the same sensory distribution. Under immunocompetent conditions, the infection usually does not spread beyond the anatomic distribution or outside the vicinity of a single dorsal root ganglion. Primary infection commonly presents during the second and third decades of life; however, reactivation can occur at any time by retrograde transmission, resulting in human encephalitis [2].

Prompt clinical recognition is important to prevent progressive brain tissue damage, hemorrhagic changes, and worsening encephalitis. Diagnosis is usually confirmed through an extensive evaluation, including a thorough clinical examination with attention to findings of mental status changes, analysis of cerebrospinal fluid (CSF) analysis, electroencephalogram (EEG) testing, and neuroimaging findings. Early brain imaging may reveal changes which may mimic as cerebral ischemic lesions and may mislead the diagnosis. Once HSVE is suspected, high-dose acyclovir should be started immediately before lumbar puncture (LP), and only stopped once a definitive alternate diagnosis has been established. Early intervention with high-dose intravenous (IV) acyclovir has been shown to improve neurological outcomes in these patients [3].

Herein, we report a case with HSVE presenting atypically as a right middle cerebral artery ischemic stroke.

## Case presentation

A 76-year-old woman was transferred to our facility for acute right middle cerebral artery (MCA) stroke. She initially presented with headache, left sided weakness, intermittent fever, and generalized malaise. She also developed laceration on the back of head due to syncopal attack. Prior to presentation, she had fever, productive cough, and dyspnea and was treated with antibiotics for pneumonia. Her past medical history included hypertension, atrial fibrillation, congestive heart failure, and chronic obstructive lung disease.

At an outlying facility emergency room (ER), her physical exam was concerning for altered mental status, left-sided weakness, and mild respiratory distress. No meningeal signs were observed. Her laboratory findings included mild leukocytosis. All cultures remained negative. Head and chest imaging were unrevealing. A lumbar puncture (LP) could not be performed because of her elevated international normalized ratio (INR) due to regular warfarin use for atrial fibrillation. She was treated empirically with acyclovir, ceftriaxone, and vancomycin. Her mental status worsened. A repeat computed tomography (CT) scan demonstrated a stroke within the medial right temporal lobe. She was transferred immediately to our tertiary care for urgent magnetic resonance imaging (MRI) and a higher level of care. 

**Figure 1:**
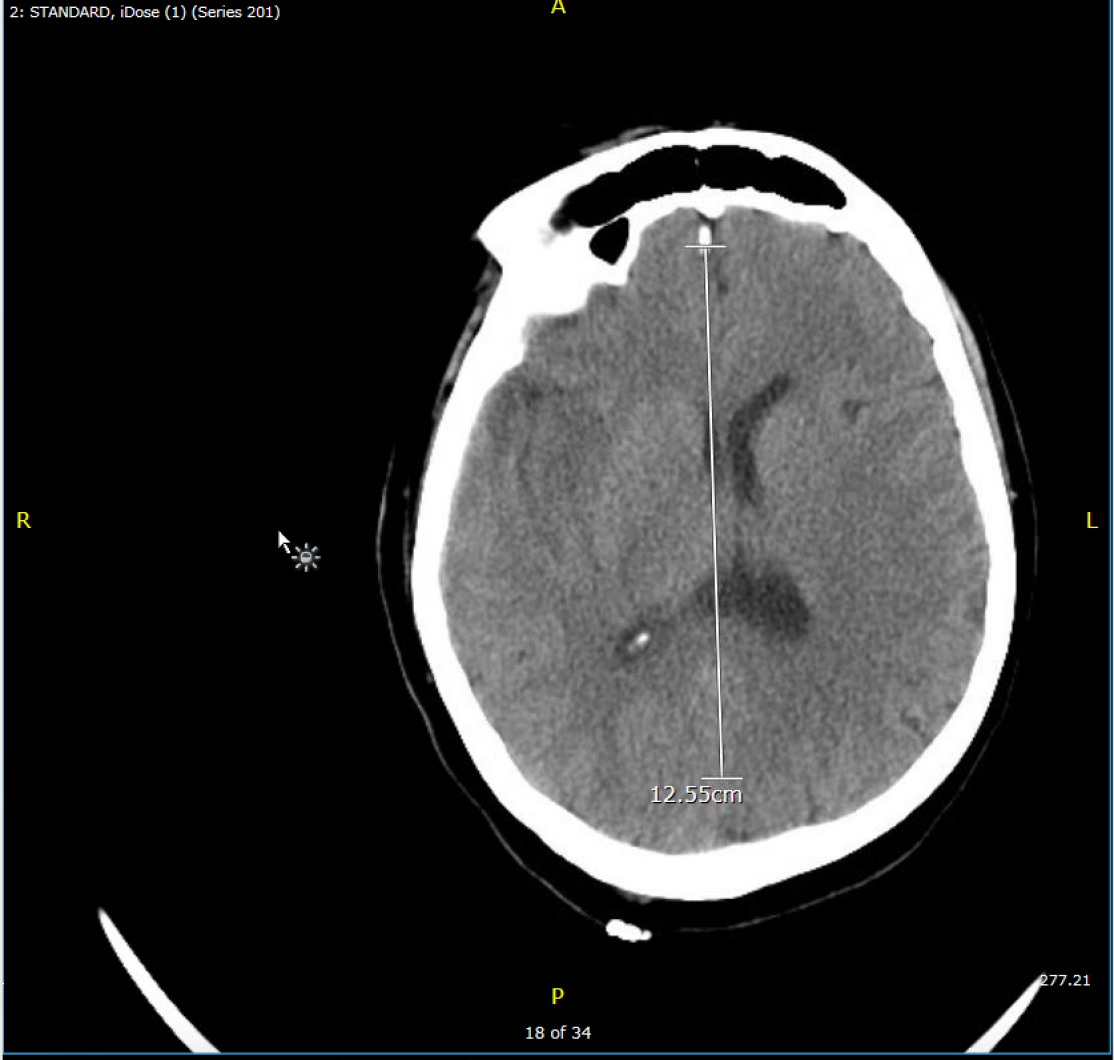
Head computed tomography (CT) scan CT of the head shows a significant acute right middle cerebral artery infarct with significant gyral swelling, mass effect, loss of gray/white differentiation in right medial temporal/insular cortex, and an external capsule with a leftward midline shift of 4 mm.

**Figure 2:**
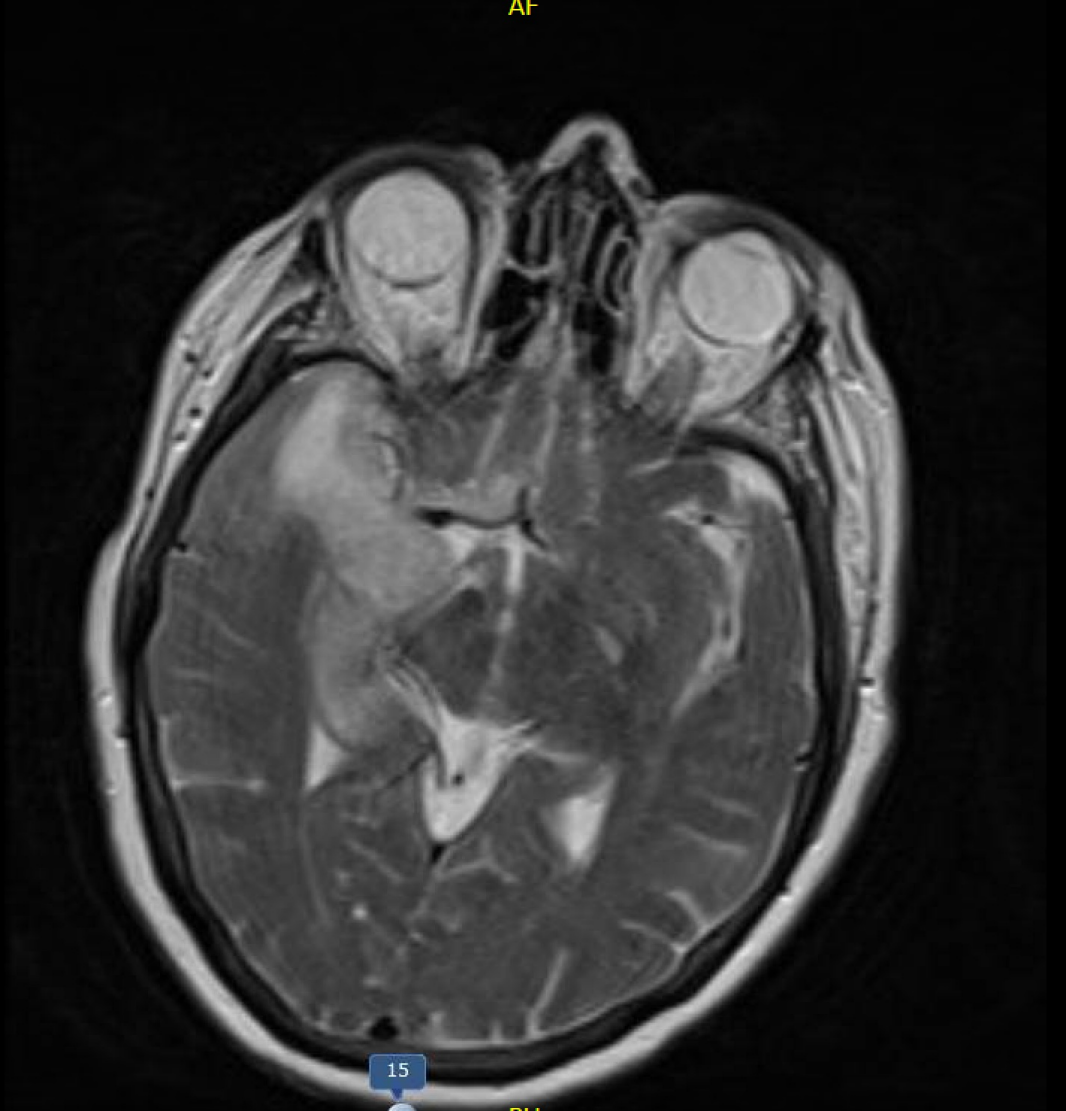
Magnetic resonance imaging (MRI) of the head MRI of the head shows an abnormality of the right temporal lobe, insular cortex, and external capsule regions with a left midline shift of 4 mm

At the time of presentation to our hospital, she was found to have an altered mental status with left-sided hemiparesis. She was febrile, tachypneic, and tachycardiac with elevated blood pressures. A repeat CT scan showed an acute right middle cerebral artery (MCA) infarct associated with significant gyral swelling, causing a left midline shift of 4 millimeters (mm) (Figure 1). A brain magnetic resonance imaging (MRI) (Figure 2) confirmed CT findings. Computed tomography angiography (CTA) did not show any acute intracranial arterial abnormality. Tissue plasminogen activator (TPA) was not administered as the patient was out of the thrombolytic window.

Our working differentials included acute ischemic stroke and herpes encephalitis. Due to the patient's atypical presentation of altered mental status, headache, left-sided weakness, syncopal episode, and radiographic findings of stroke, an electroencephalogram (EEG) was performed, which showed a high epileptogenic potential in the right hemisphere with poor localization. Hypertonic saline was started to reduce brain edema, along with IV acyclovir 10 mg/kg thrice a day and levetiracetam. As the edema subsided and INR normalized, an LP was performed. Polymerase chain reaction analysis of CSF was positive for herpes simplex virus (HSV) type one, supporting the diagnosis of herpetic encephalitis.

A video EEG to check therapeutic and prognostic response after treatment was done. On the fifth day, the patient showed significant clinical improvement. 

## Discussion

Herpes simplex viral encephalitis (HSVE) is the most frequent cause of sporadic fatal encephalitis in the Western world, with an incidence of one case per million per year [1]. Nevertheless, the condition is rare and presents with non-specific symptoms; it has a varied nature of presentation making a diagnosis of HSVE challenging in both adults and children. The virus infects the sensory branch of the lingual nerve, then ascends to the trigeminal ganglion and remains latent. Reactivation can result in fulminant hemorrhagic necrotizing encephalitis. The clinical manifestation typically presents with fever, headache, confusion, and focal or generalized seizures [2]. Around 90% of the patients have fever and abnormal mental status as the primary signs and symptoms of HSVE. Vomiting, nausea, pseudomeningitis, and seizures occur in about 50% - 60% of patients and focal neurological deficits appear in 30 - 50% [1].

Townend, et al. also reported a similar case regarding an elderly female with confusion and reduced mobility. The differentials included delirium, sepsis, and possibly stroke. She was febrile on presentation and had a prodrome of three days. With non-specific early brain CT appearances, she was misdiagnosed as pneumonia with stroke instead of HSVE [4]. AbdulJabbar and colleagues reported the case of a 17-year-old girl who presented with headache and acute onset hemiparesis preceded by classical HSVE presentation, including fever, altered mental status, and seizures. Similar to our patient, her diagnosis was based on MRI and positive titers of antibodies against herpes simplex type one in CSF analysis. This puts it in the differential diagnosis of acute stroke in young patients, even in the absence of encephalitic features, and also emphasizes that early MRI examination could be of great help in delineating the pathology at presentation [5].

The isolation of the HSV in the CSF has a diagnostic value in HSVE. The CSF analysis usually demonstrates pleocytosis and predominant lymphocytosis with elevated protein. The serological tests immunoglobulin M (IgM) and immunoglobulin G (IgG) are sensitive to detect low amounts of antibodies from spinal fluid. The gold standard to confirm an HSVE diagnosis is to demonstrate HSV deoxyribonucleic acid (DNA) in the cerebrospinal fluid using polymerase chain reaction (PCR) [2]. A positive CSF analysis also played a role that led to the confirmation of HSVE in our case.

HSV has a high affinity for limbic systems with bilateral or asymmetrical involvement. The limbic system is an integrated structure of the brain involved in memory function; as such, the patient may also present with memory impairment and disorientation to place, time, and person [6]. Pirasath, et al. reported a patient encounter of an elderly female with a history of fever, alongside a headache for 10 days and a single episode of seizure on the day of admission. She denied weakness; however, she was confused and disorientated to place, time, and person with receptive aphasia [4]. Receptive aphasia is also a common presentation of HSVE, occurring in about 33 - 65% of the cases on retrospective analysis, giving the impression of a stroke on initial evaluation [7]. However, our patient had no obvious speech deficits due to its involvement in the right hemisphere.

On MRI, lesions due to HSVE are characterized by restricted diffusion, which results from cytotoxic edema. This can potentially be mistaken for middle cerebral artery (MCA) infarct, especially when involving only one vascular distribution. HSV has a predilection for the temporal lobes, insula, and cingulate. It often can be seen as increased T2-weighted fluid-attenuated inversion recovery (T2/FLAIR) signal intensity, which is highly specific. However, the basal ganglia are typically spared in HSVE, which helps to distinguish it from an MCA infarction. In their case report, Shalchi, et al. also mentioned misdiagnosis of HSVE as an ischemic stroke based on right-sided frontal and temporal hypodensities noted radiologically [8].

Electroencephalography (EEG) is a nonspecific but sensitive tool that shows abnormalities in the medial temporal and inferolateral frontal lobes with bilateral but typically asymmetrical involvement. Because of this, EEG can play a significant role in the diagnosis as well as the progression of HSVE [3]. In a separate retrospective study, EEG was able to localize HSVE earlier in around 80% of patients [9]. Abdelmalik, et al. have reported the case of an elderly lady who presented with acute onset global aphasia and right hemiparesis in the absence of a prodromal phase, initially diagnosed as a proximal left middle cerebral artery (MCA) stroke. However, CT perfusion and CTA did not show evidence of a proximal vessel occlusion. Furthermore, MRI brain also did not demonstrate any areas of restricted diffusion. EEG demonstrated left temporal periodic lateralized epileptiform discharges (PLEDs) supporting a diagnosis of HSVE [9].

HSVE is associated with significant morbidity and mortality, particularly when appropriate management is delayed. Acyclovir is a well-tolerated medication and is very effective in inhibiting viral replication and delaying axonal spread to newly infected regions in the brain. It has become an established practice that treatment for HSVE is started on clinical suspicion. Untreated, the mortality of HSVE approaches 70% [4]. Patients have been shown to have a poor outcome after HSVE if treatment with acyclovir is delayed by two days after admission, if the Glasgow coma score is 6 or below, and age is higher than 30. However, 38% of patients who received acyclovir without any delay had a good recovery with no residual neurological deficits [10].

Often, the most common reason for the delay is a failure to consider HSVE among the initial diagnostic possibilities. Frequently, ischemic stroke is first to be ruled out, particularly when a patient presents with acute onset focal deficits and absent fever or prodrome. Many institutions obtain immediate CT perfusion, along with a non-contrast CT head, in the triage of acute stroke patients. With a negative head CT and pending MRI, physicians may suspect HSVE and an immediate EEG or CSF analysis should also be considered to aid in diagnosis.

## Conclusions

Herpes simplex viral encephalitis (HSVE) is associated with a 70% mortality if untreated. Patients have shown to have a poor outcome after HSVE in those in whom treatment with acyclovir is delayed by more than two days after admission. Immediate correlation of imaging studies with the clinical picture is important. This case highlights the importance of a multidisciplinary approach and the need for increased awareness of an atypical presentation of HSVE among emergency physicians, neurologist, intensivists, and radiologists, given that the early spectrum of clinical and CT findings can mimic sepsis or stroke.
